# The 3 31 Nucleotide Minihelix tRNA Evolution Theorem and the Origin of Life

**DOI:** 10.3390/life13112224

**Published:** 2023-11-19

**Authors:** Lei Lei, Zachary Frome Burton

**Affiliations:** 1 School of Biological Sciences, University of New England, Biddeford, ME 04005, USA; llei@une.edu; 2 Department of Biochemistry and Molecular Biology, Michigan State University, East Lansing, MI 48824, USA

**Keywords:** accretion model, convergent evolution model, divergent evolution model, folding of first proteins, genetic code evolution, origin of life, pseudosymmetry, ribozyme/primitive catalyst RNA ligation, type I and type II tRNA evolution

## Abstract

There are no theorems (proven theories) in the biological sciences. We propose that the 3 31 nt minihelix tRNA evolution theorem be universally accepted as one. The 3 31 nt minihelix theorem completely describes the evolution of type I and type II tRNAs from ordered precursors (RNA repeats and inverted repeats). Despite the diversification of tRNAome sequences, statistical tests overwhelmingly support the theorem. Furthermore, the theorem relates the dominant pathway for the origin of life on Earth, specifically, how tRNAomes and the genetic code may have coevolved. Alternate models for tRNA evolution (i.e., 2 minihelix, convergent and accretion models) are falsified. In the context of the pre-life world, tRNA was a molecule that, via mutation, could modify anticodon sequences and teach itself to code. Based on the tRNA sequence, we relate the clearest history to date of the chemical evolution of life. From analysis of tRNA evolution, ribozyme-mediated RNA ligation was a primary driving force in the evolution of complexity during the pre-life-to-life transition. TRNA formed the core for the evolution of living systems on Earth.

## 1. Evolution of TRNA

A number of models have been advanced to describe tRNA evolution. We have advanced the 3 31 nt minihelix theorem [1,2,3]. To support minihelix replication in pre-life, 3 31 nt minihelices were joined by ligation. The D loop 31 nt minihelix had the sequence GCGGCGG_UAGCCUAGCCUAGCCUA_CCGCCGC (the _ separates distinct sequence features). The D loop minihelix is a 7 nt GCG repeat (5′-acceptor stem) linked to a 17 nt UAGCC repeat (D loop minihelix core) linked to a 7 nt CGC repeat (3′-acceptor stem). The anticodon loop and the T loop 31 nt minihelices were probably initially identical, with the sequence GCGGCGG_CCGGG_CU/???AA_CCCGG_CCGCCGC (/ indicates a U-turn; ? indicates A, G, C or U (the pre-life base remains unknown)). This is a 7 nt GCG repeat (5′-acceptor stem) linked to a 17 nt stem-loop-stem (CCGGG_CU/???AA_CCCGG) linked to a 7 nt CGC repeat (3′-acceptor stem). The only pre-life sequence ambiguities are in the 7 nt CU/???AA loops, not in the stems. After LUCA (the last universal common (cellular) ancestor), the dominant anticodon loop sequence was CU/BNNAA or CU/BNNGA (B indicates G, C or U, not A; N indicates A, G, C or U), and the dominant T loop sequence was UU/CAAAU. Moreover, 7 nt loop sequences for the anticodon loop and the T loop were separately selected in evolution because of their different placements within tRNA. The anticodon loops were selected to generate the distinct anticodons required for coding. The T loop sequence was selected to form the tRNA elbow at which the D loop and the T loop interact to form the L-shaped tRNA fold [4]. T loop C3 (tRNA-56) forms a bent Watson–Crick base pair to a D loop G (G19 in standard tRNA numbering; for historical reasons, standard tRNA numbering breaks down in the D loop and V loop). D loop G18 intercalates between T loop A4 (tRNA-57) and A5 (tRNA-58), lifting T loop A6 (tRNA-59) and U7 (tRNA-60) out of the loop. G18 was initially an A, as part of the third UAGCC repeat, but was mutated to G to support D loop–T loop interactions at the elbow. This G to A transition in the D loop is one of the very few systematic sequence changes to support the tRNA fold versus the parental 31 nt minihelix folds. In summary, tRNA evolved in pre-life from ligation of 3 31 nt minihelices followed by internal RNA processing (described below). The 31 nt minihelices that were utilized were of two distinct sequences (D loop and anticodon/T loop). Pre-life tRNA sequences were comprised of RNA repeats and inverted repeats. Post-LUCA, the dominant tRNA anticodon and T loop sequences are known because these sequences have been conserved for ~4 billion years in living organisms. Post-LUCA tRNA sequences can be recovered as typical tRNA sequences from a tRNA database [5]. The 3 31 nt minihelix tRNA evolution theorem, therefore, can readily be confirmed from typical tRNA diagrams of ancient Archaea (i.e., Pyrococcus furiosis, Sulfolobus solfataricus, Aeropyrum pernix, Staphylothermus marinus).

Alternate models for tRNA evolution are of the following types: (1) convergent models [6,7,8,9,10,11,12,13,14,15,16,17]; (2) accretion models [6,7,8,9,10,11,12,13,14,15,16,17,18]; (3) 2 minihelix models [6,11,12,14,16,17]; and (4) highly theoretical models [7,8,10,19,20]. First of all, no convergent model can rationally apply to tRNA evolution. To evolve, tRNA requires a divergent evolution model, in which tRNAomes (all of the tRNAs of an organism) diverged from pre-life type I and type II tRNAs by repeated duplications and re-purposing events. In a convergent model, multiple small tRNA fragments must converge on the same homologous sequence, conformation and form. For tRNA, such a convergent evolution model is untenable, and all convergent models are accretion models because bases must be added and/or subtracted to gain the final tRNA form. Because pre-life tRNAs were generated from completely ordered sequences (RNA repeats and inverted repeats), no accretion or convergent model can be descriptive [2,3,21,22,23,24,25]. Accretion models have reasonably been applied to describe later stages of rRNA evolution [26,27,28]. For tRNA, by contrast to rRNA, accretion models are convergent models that demand convergence of multiple pre-tRNAs (tRNA fragments) to similarly structured and ordered RNA sequences. For initial tRNA evolution, no accretion model is reasonable. 

Multiple 2 minihelix models have been advanced for tRNA evolution. All of these models are convergent and accretion models. One disqualifying objection to 2 minihelix models is that 2 minihelix models are inconsistent with the homology of the anticodon stem-loop-stem and the T stem-loop-stem [3]. Because the 17 nt anticodon and the 17 nt T stem-loop-stems are clearly homologous (i.e., by inspection), no 2 minihelix model can possibly be correct. In a 2 minihelix model, the anticodon and T stem-loop-stems would be required to converge on a homologous conformation (i.e., compact 7 nt U-turn loop) and homologous sequence. 

The uroboros (hoop snake with mouth grasping tail) model for tRNA evolution was computer-generated and remains highly theoretical [9,10]. In the uroborous vision, numerous 22 nt covalently closed tRNA rings converged and accreted to homologous and ordered tRNA forms. The uroboros model cannot be correct for tRNA evolution. Some authors employ convergent and accretion tRNA evolution models without realizing their obvious mistake [29,30,31,32,33,34].

## 2. The Evolutionary History of Life on Earth

Top-down strategies to describe pre-life evolution have the potential advantage of identifying dominant, successful pathways because only routes that survived can be detected [1,2]. The 3 31 nt minihelix theorem relates a top-down, sequence-based argument for two stages of pre-life evolution: (1) polymer world and (2) 31 nt minihelix world. The central focus of the top-down argument is that highly ordered tRNA sequences have been conserved from pre-life to life and maintained since LUCA. Bottom-up strategies would be attempts to create life or a potential pre-life chemical state in vitro [35,36,37,38,39,40,41]. Bottom-up strategies result in novel chemistry but may represent evolutionary dead-ends. When top-down and bottom-up descriptions can be linked, understanding of the pre-life-to-life transition is significantly enriched. Because tRNA sequences are ordered, the 3 31 nt minihelix tRNA evolution theorem describes hundreds of millions of years of pre-life chemical evolution.

**Figure 1:**
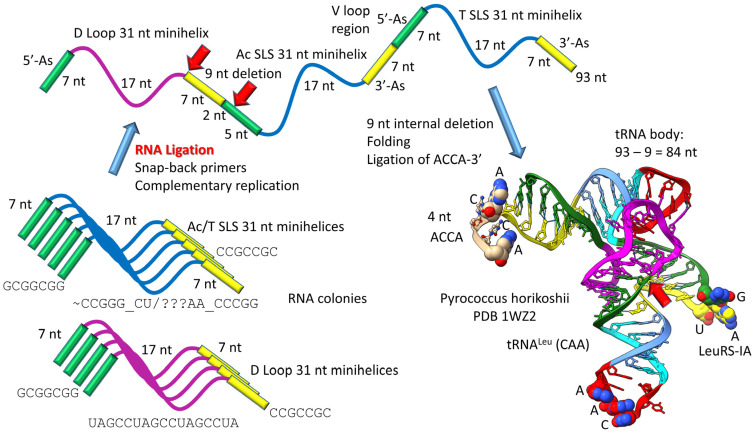
Type II tRNA evolved via RNA ligation and a 9 nt internal deletion within ligated 3′- and 5′-acceptor stems [42]. Also, 3 31 nt minihelices (one D loop minihelix (magenta 17 nt core) and two anticodon stem-loop-stem minihelices (blue 17 nt core)) were fused by ligation for minihelix replication. The 93 nt tRNA precursor was processed by an internal 9 nt deletion (see below) within fused 3′-acceptor (yellow) and 5′-acceptor (green) stems. In the type II tRNA structure, the red arrow indicates the fusion of the magenta segment (17 nt D loop minihelix core; UAGCC repeat) and the green segment (5′-acceptor stem fragment; initially GGCGG). Abbreviations: SLS) stem-loop-stem; Ac) anticodon. Molecular graphics were created using ChimeraX [43].

**Figure 2:**
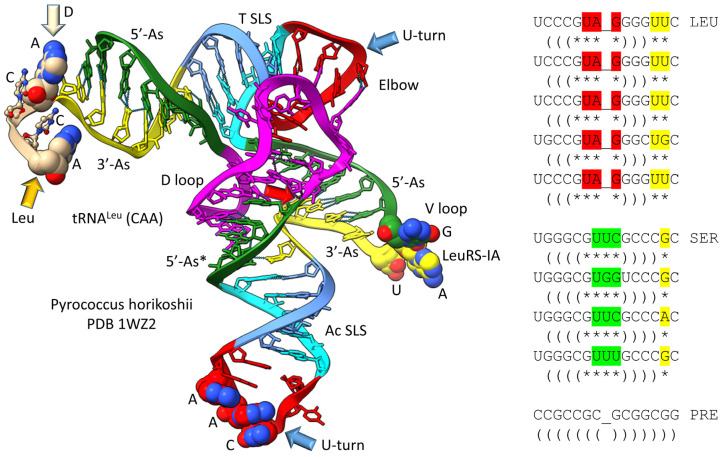
Type II tRNA resulted from failure to process a 14 nt V loop (initially a 7 nt 3′-acceptor stem ligated to a 7 nt 5′-acceptor stem) rather than by accretion. Colors: green) 5′-acceptor stem and 5′-acceptor stem fragment; magenta) 17 nt D loop core; cyan) 5′-anticodon and T stem; red) anticodon and T loops; cornflower blue) 3′-anticodon and T stem; and yellow) 3′-acceptor stem. Arrow colors: blue) U-turns; red) processing site in evolution; light yellow) discriminator base (D); and gold) site of amino acid placement. The structure is of an unmodified Pyrococcus horikoshii tRNA^Leu^ in complex with LeuRS-IA. At the right of the figure are tRNA^Leu^ and tRNA^Ser^ V loops from Pyrococcus furiosis, an ancient Archaeon. Colors: red) V loop UAG that binds LeuRS-IA in tRNA^Leu^ recognition in P. furiosis [44]; yellow) unpaired bases just 5′ of the Levitt base; and green) tRNA^Ser^ bases at the 3′-end of the V loop. PRE indicates an initial pre-life sequence. Parentheses indicate stems; * indicates unpaired bases.

**Figure 3:**
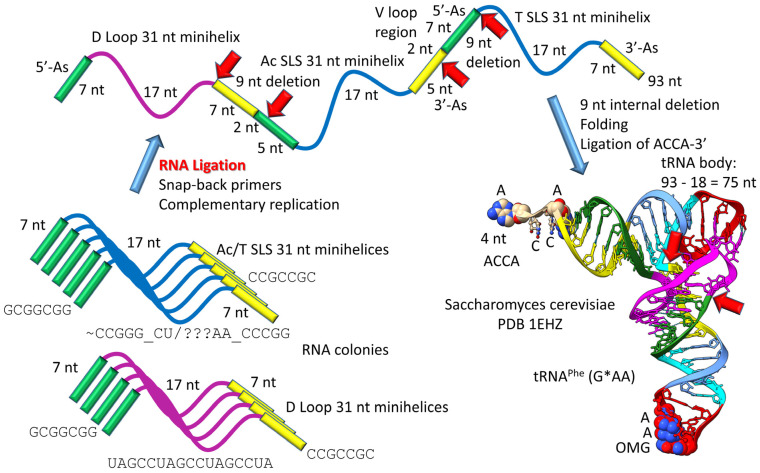
Evolution of type I tRNA via RNA ligation and two related, internal 9 nt deletions. Colors and arrow colors are as in Figure 1 and Figure 2. G* (OMG) is 2′-O-methyl-G. Also, 9 nt internal deletions generate a magenta-green fusion and a yellow-cyan fusion (red arrows).

**Figure 4:**
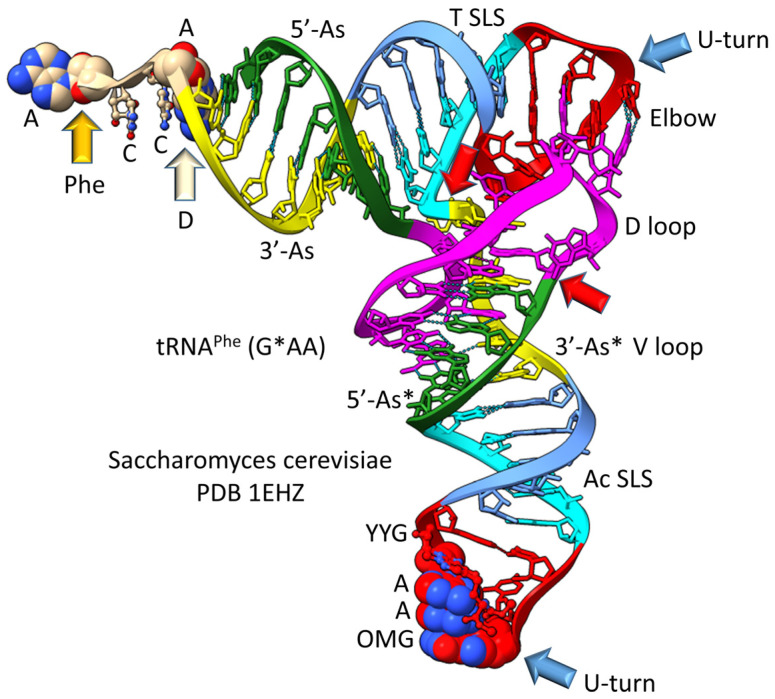
Type I tRNA. Colors and arrow colors are as in Figure 1 and Figure 2. G* (OMG) is 2′-O-methyl-G. YYG is Wy-butosine [46]. The V loop (3′-As*; yellow) is fused to the cyan (5′-T stem), in slight contrast to type II tRNA processing (Figure 1 and Figure 2).

The history of the evolution of life on Earth embedded in the tRNA sequence is shown in Figure 1, Figure 2, Figure 3 and Figure 4. Remarkably, tRNA evolution reduces to a simple sequence puzzle that anyone who can read a four-letter code can solve or verify. Figure 1 and Figure 2 explain type II tRNA evolution [42]. Figure 3 and Figure 4 explain type I tRNA evolution, which is a very similar process [3,21,23,24,25]. Both type II and type I tRNAs evolved from the same 93 nt tRNA precursor. Type II tRNA evolution converted the 93 nt precursor, which was formed by ligation of 3 31 nt minihelices with two distinct 17 nt core sequences, into type II tRNA through a single internal 9 nt deletion (Figure 1). Generation of a pre-life type II tRNA required only a single internal 9 nt deletion within ligated 3′- and 5′-acceptor stems, as shown (red arrows). In Figure 1, 7 nt of a 3′-acceptor stem (yellow) and 2 nt of a 5′-acceptor stem (green) were deleted, leaving a 5 nt fragment of a 5′-acceptor stem (initially GGCGG; green). The internal deletion fused a magenta segment (a 17 nt UAGCC repeat) to a green segment (a 5′-acceptor stem fragment; 5 nt). The initial type II tRNA body (lacking the 4 nt ACCA adapter) was 93 − 9 = 84 nt (so the total type II tRNA length was initially 88 nt). 

The original pre-life type II tRNA V loop (V for variable) was a 7 nt 3′-acceptor stem (yellow) ligated to a 7 nt 5′-acceptor stem (green) (initially, CCGCCGC_GCGGCGG) [42]. Because the pre-life V loop sequence was self-complementary along its entire length, the V stem-loop-stem evolved to sequences such as those found in archaeal tRNA^Leu^ and tRNA^Ser^. V loops for tRNA^Leu^ and tRNA^Ser^ from Pyrococcus furiosis were selected to be distinct (i.e., for separate discrimination by LeuRS-IA and SerRS-IIA) and are shown in the right panel of Figure 2, compared to the initial pre-life V loop [5]. V loops for tRNA^Leu^ are numbered V_1_ to V_14_ (14 nt was the primordial length). UV_1_ forms a wobble pair with G26 (standard tRNA numbering). CV_14_ forms a reverse Watson–Crick pair to G15 (the Levitt base pair; see below). Two unpaired bases are found at V_12_ and V_13_ (UU or UG). The number of unpaired bases just 5′ to the Levitt base pair determines the trajectory of the V stem-loop-stem from the tRNA body. V_6_-UAG-V_8_ binds the tRNA^Leu^ charging enzyme LeuRS-IA (leucine aminoacyl–tRNA synthetase; structural class and subclass IA) [44]. None of the corresponding tRNA^Ser^ V loop sequences are sufficiently similar to UAG (UUC, UGG, UUU; green shading), so LeuRS-IA will not bind to a tRNA^Ser^ V loop, even if the loop could be accessed by LeuRS-IA. For P. furiosis, the length of the tRNA^Ser^ V loop expanded from 14 to 15 nt. In tRNA^Ser^, only 1 nt is unpaired just 5′ of the Levitt base pair. The tRNA^Ser^ V stem-loop-stem, therefore, has a different trajectory from the tRNA^Ser^ body compared to tRNA^Leu^. SerRS-IIA binds the V stem [45], not the V loop, so SerRS-IIA is dependent on the trajectory of the V stem-loop-stem from the tRNA^Ser^ body compared to the distinct trajectory of the V stem-loop-stem of tRNA^Leu^. In summary, type II tRNA evolution is described to the last nucleotide by the 3 31 nt minihelix theorem. The longer V loop in type II tRNAs was initially generated by a failure to process ligated 3′- and 5′-acceptor stems, rather than by insertion of bases (accretion).

Most tRNAs are type I, with a shorter V loop (i.e., 5 nt) compared to type II tRNAs (initially 14 nt). Notably, the 3 31 nt minihelix theorem describes type I tRNA evolution to the last nucleotide (Figure 3 and Figure 4). In Figure 3, type I tRNA evolution is shown from the same 93 nt tRNA precursor. In this case, two closely related 9 nt deletions occurred within ligated 3′- and 5′-acceptor stems. The more 5′ internal 9 nt deletion was identical to the processing event that generated type II tRNAs (Figure 1). The more 3′ 9 nt deletion removed 7 nt of the 5′-acceptor stem (green) and 2 nt of the 3′-acceptor stem (yellow), leaving 5 nt of the 3′-acceptor stem (initially CCGCC; yellow). Thus, in type I tRNA evolution, CCGCC (yellow) was linked to CCGGG (cyan). In type I tRNA, the more 5′ and 3′ 9 nt deletions within ligated acceptor stems are closely related. If the deletions occurred on complementary RNA strands, the more 3′ 9 nt deletion was identical to the more 5′ 9 nt deletion. Below, we suggest a ribozyme/primitive catalyst processing mechanism to describe the internal 9 nt deletions. In summary, type II and type I tRNAs evolved by a common mechanism that involved ligation of the same 3 31 nt minihelices to form the same 93 nt tRNA precursor followed by internal processing and deletion of complementary 9 nt segments. The original type I tRNA was 93 − 18 = 75 nt (tRNA body) plus the 4 nt ACCA adapter sequence (so 79 nt total). It is likely that the first tRNAs were mixtures of type II and type I tRNAs. 

## 3. The 3 31 nt Minihelix Theorem

**Figure 5:**
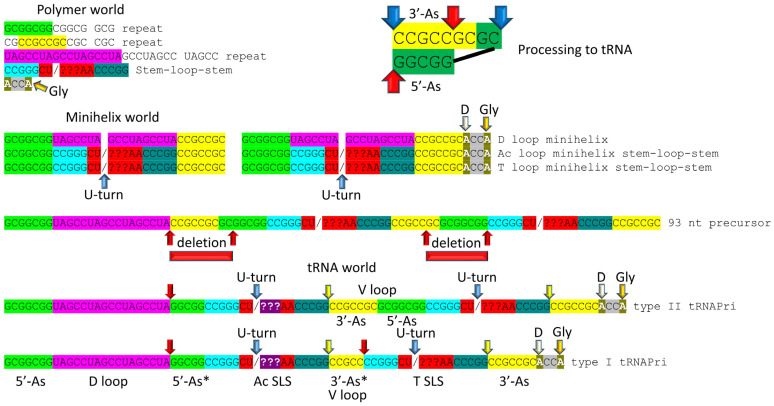
The 3 31 nt minihelix theorem, and evolution of tRNA world from polymer world and 31 nt minihelix world. The inset describes the 9 nt deletions to generate tRNAs: the more 5′ processing event involves deletion between the blue arrows; the more 3′ processing event (type I tRNA only) involves deletion between the red arrows. Internal deletions were at stem-loop junctions. Colors and arrows are consistent with previous figures. Yellow arrows mark the cornflower blue-yellow junction, indicating the degree of order in tRNA assembly.

The 3 31 nt minihelix theorem is shown in Figure 5 as a linear sequence. The inset in the figure shows a mechanism to generate the 5′- (type I and type II tRNAs) and 3′- (type I tRNA only) acceptor stem fragments found in tRNAs. The proposed mechanism involved a ribozyme/primitive catalyst endonuclease to cleave RNAs at stem-loop boundaries followed by RNA ligation. RNA stem-loop-stems cannot maintain a 2 nt loop because a 2 nt loop is too constrained, so 4 nt loops were the expected substrates for processing (as shown). 

Embedded in tRNA sequences is evidence for the minihelix world and, before that, the polymer world [1,2,21]. Surprisingly, precursor tRNA sequences from pre-life were highly ordered: RNA repeats and inverted repeats [3,24,25,42]. Initially, this was a shock to us because we thought tRNA was generated by a chaotic process. Inspection of tRNA sequences, however, shows that pre-life tRNA precursors were ordered. Because tRNAs were generated from ordered sequences, pre-life tRNA precursors were arranged as RNA repeats and inverted repeats, as shown. Analysis of tRNA sequences, therefore, reveals a history of pre-life worlds on Earth. It is not reasonable to consider that ordered tRNA precursors were generated by any chaotic process. We stress that these recovered sequences from living organisms are fossils from the pre-life-to-life transition on Earth ~4 billion years ago.

## 4. Polymer and 31 nt Minihelix Worlds

In pre-life, the polymer world, minihelix world and tRNA world were overlapping and more complex than can now be described just from the tRNA sequence. The surviving history was limited to tRNA sequences. This is a limitation of top-down and sequence-based analyses of pre-life. The polymer world included GCG repeats, CGC repeats and UAGCC repeats, which are sequences found in tRNAs. We posit that the polymer world included ACCA as the most primitive RNA-amino acid adapter. Attaching glycine at the 3′-end, ACCA becomes ACCA-Gly, which can be used for polyglycine synthesis. A GCG repeat includes multiple CGGC sequences, which can anneal with ACCA-Gly for polyglycine synthesis. Hydration–dehydration cycles may have supported polyglycine synthesis with multiple ACCA-Gly immobilized in proximity [38,47]. Phase separations supported by water, supercritical CO_2_ and lipid within the Earth’s crust under high pressure may also have contributed to polypeptide polymerization reactions [39,40,41].

**Figure 6:**
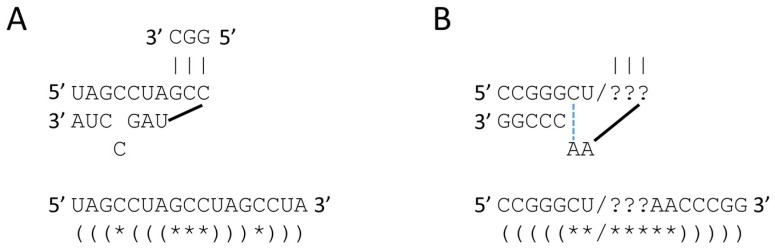
Features of polymer world. (**A**) The D loop minihelix core could function as a primitive translational adapter to recognize codon GGC. (**B**) The anticodon and T stem-loop-stems could function as a translational adapter. The dotted blue line indicates a Hoogsteen A–C pair that stabilizes the U-turn loop. Ligation of 3′-ACCA-Gly converted these sequences into primitive translational adapters in the pre-life world. Parentheses indicate paired bases. Asterisks indicate loop bases.

Figure 6 shows some of the features expected in polymer and 31 nt minihelix worlds. Figure 6A shows that the D loop minihelix core is self-complementary and can present anticodon GCC to pair codon GGC. GGC is found in the GCG repeat identified in the polymer world from analysis of tRNA sequences (Figure 5). Figure 6B shows the anticodon and T stem-loop-stem sequences. The codon recognized would depend on the ??? sequence (i.e., anticodon GCC would recognize codon GGC). If ACCA-Gly were ligated at the RNA 3′-ends, these sequences could be utilized for polyglycine synthesis. In pre-life, the stem-loop-stem sequence CCGGG_CU/???AA_CCCGG indicates that some form of complementary replication was present in the polymer world. Also, a stem-loop-stem can function as a primer for complementary replication. We posit that the anticodon stem-loop-stem was the most central intellectual property in pre-life on Earth. The 7 nt loop sequence (CU/???AA) allowed tRNA to learn to code.

We posit that the minihelix world evolved and was selected alongside the polymer world as an improved means to generate polyglycine [37,48]. When we invoke polyglycine or GADV world, we do not mean to imply that the synthesis of these sequences was particularly accurate. Other available amino acids could have been incorporated, as well. Translational fidelity coevolved with the genetic code only as sequence-dependent proteins became more strongly protected by selection. Judging from the tRNA sequence, the minihelix world was the clear precursor of the tRNA world because tRNA was evolved by ligation of 3 31 nt minihelices of almost completely known sequence (Figure 1, Figure 2, Figure 3, Figure 4 and Figure 5). To generate the minihelix world from the polymer world required a small number of ribozymes/primitive catalysts, most or all of which have been generated by scientists in vitro. Similarly, a small set of ribozymes/primitive catalysts would be sufficient to convert the minihelix world into the tRNA world. Most likely, these conversions could be reproduced in vitro.

Organisms have lived in the tRNA world for about 4 billion years. The advantage of the tRNA world over the polymer and minihelix world was, initially, that tRNA evolved and was selected as an improved means to synthesize polyglycine as a component of protocells [1,2]. From the tRNA–polyglycine world, the tRNA–GADV world emerged [36,49,50,51,52]. From an 8 amino acid stage (i.e., GADVLSER), the genetic code and sequence-dependent proteins emerged [1,2,21]. Thus, no chicken and egg problem need be invoked in the evolution of the genetic code because the system did not need the foresight that it would eventually encode sequence-dependent proteins. Polyglycine and GADV polypeptides were selected for coacervate functions (i.e., supporting membrane-less organelles in protocells) [1,2,53]. With added complexity, the genetic code and sequence-dependent proteins evolved and were selected and coevolved with tRNAomes [21,23]. Once the genetic code evolved, living systems began.

## 5. Arguments for and against the 3 31 nt Minihelix Theorem

For reasons that we do not understand, the 3 31 nt minihelix theorem, which is fully supported by the tRNA sequence, has not gained universal acceptance. The 3 31 nt minihelix theorem is important because it relates the history of the pre-life-to-life transition on Earth. We understand that there are competing tRNA evolution models, but alternate models are falsified. The competing models are all chaotic, convergent, accretion and 2 minihelix models. None of these models can possibly be reasonable. 

Statistics strongly favor the 3 31 nt minihelix theorem (Table 1) [24]. Remarkably, every feature of the theorem was predicted and is supported by statistical analysis. As the analysis was performed, a *p*-value of 0.001 indicated a similar sequence with a 1 in 1000 chance of being due to random chance. So, a *p*-value of 0.001 indicates homology. A *p*-value approaching 1 indicates sequences are not homologous. A *p*-value of <0.05 would indicate similarity and probable homology. First of all, as expected, the 5′-acceptor stem is apparently homologous to the complement of the 3′-acceptor stem with which it pairs. Also, as expected, the 3′-acceptor stem is homologous to the complement of the 5′-acceptor stem. The demonstrated complementarity of the 5′- and 3′-acceptor stems appears to partly verify the statistical test. The 5′-acceptor stem fragment tests as homologous to the 5′-acceptor stem, positions 3–7. The 3′-acceptor stem fragment (type I V loop) tests as homologous to the 3′-acceptor stem, positions 66–70, as expected from the theorem. The 17 nt anticodon stem-loop-stem tests as homologous to the 17 nt T stem-loop-stem, as anticipated from the theorem. As expected, neither the 17 nt anticodon stem-loop-stem nor the 17 nt T stem-loop-stem test is homologous to the 17 nt D loop core (initially, a UAGCC repeat). Both the type II tRNA^Leu^ and tRNA^Ser^ V loops test as homologous to a 3′-acceptor stem ligated to a 5′-acceptor stem (Table 1) [42]. In summary, every aspect and prediction of the 3 31 nt minihelix theorem is reinforced by statistical tests. The theorem completely describes the evolution of type II and type I tRNAs to the last nucleotide. 

**Figure 7:**
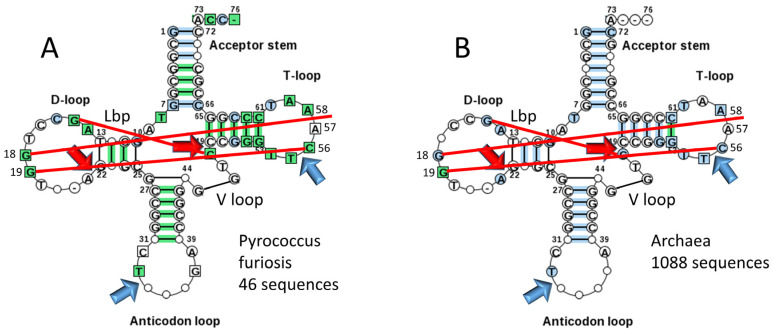
Typical tDNA diagrams for Pyrococcus furiosis (an ancient Archaeon) (**A**) and Archaea (**B**) [5]. Arrow colors: red) processing positions for evolution of type I tRNAs; and blue) U-turns. Red lines indicate: (1) the Levitt reverse Watson–Crick base pair (G15-CV_5_) (Lbp for Levitt base pair); (2) intercalation of G18 between A57 and A58 (“elbow” contact); and (3) Watson–Crick interaction of G19 and C56 (“elbow” contact) [4].

Typical tDNA sequences from the tRNA database are shown in Figure 7 for Pyrococcus furiosis (Figure 7A) and a large collection of Archaea (Figure 7B) [5]. The greener image in Figure 7A indicates a stronger consensus, indicating that some Archaea are more derived from LUCA compared to P. furiosis. We consider the ancient Archaeon P. furiosis as a reasonable model organism for LUCA. Strangely, Di Giulio has argued that the 17 nt anticodon and 17 nt T stem-loop-stems, each with a compact 7 nt U-turn loop, with the U-turn positioned between loop positions 2 and 3, are not homologs [11]. Di Giulio’s assertion is not credible. The anticodon stem-loop-stem and the T stem-loop-stem are homologous by inspection and by statistical test (Figure 7; Table 1). According to Di Giulio’s tRNA evolution model, the 17 nt anticodon stem-loop-stem and the 17 nt T stem-loop-stem must take on apparent homologous sequences and common structures by convergent evolution [12,14,54].

One might argue that acceptor stems are not based on a GCG (5′-acceptor stem) and complementary CGC (3′-acceptor stem) repeat, although inspection and statistical analyses support the sequence repeats [24]. The typical P. furiosis tRNA sequence gives the 5′-acceptor stem sequence as GCGGCGG (a perfect GCG repeat) and the 3′-acceptor stem sequence as CCGC??C (G pairs with both C and U; ? indicates that the typical base was not scored) (Figure 7A). For a larger collection of Archaea in the tRNA database, the typical 5′-acceptor stem sequence is GC?GCGG and the typical 3′-acceptor stem sequence is CCG??GC (Figure 7B). Acceptor stems diverge from a perfect GCG repeat for distinct recognition by cognate AARS enzymes. In P. furiosis, the tRNA^Ser^ D loop core begins with two perfect UAGCC repeats UAGCCUAGCC. A tRNA^Gly^ 17 nt D loop core sequence is UAGUCUAGCCUGGUCUA, which is a very close match to a UAGCC repeat [5]. The P. furiosis tRNA^Gly^ sequence is the closest to tRNA^Pri^ (the pre-life, primordial tRNA sequence; Figure 5). The closest homology of tRNA^Pri^ and tRNA^Gly^ is consistent with life evolving from a tRNA–polyglycine world [23]. Because acceptor stems and their fragments in tRNA evolved from GCG and CGC repeats and the 17 nt D loop core evolved from a UAGCC repeat, the pre-life world generated RNA repeats, and, at least in some cases, their complement (GCG and CGC repeats are complementary). Anticodon and T stem-loop-stems are snap-back primers and self-complementary at the stems. One might try to argue that the RNA repeats and homologous inverted repeats conserved in tRNAs for ~4 billion years were all caused by convergent evolution, but such an argument would be untenable. For the highly skeptical, at the very least, the 3 31 nt minihelix theorem is a remarkably good model for tRNA evolution from ~4 billion years ago. Our position is that tRNA sequences embed an unambiguous history of the most central process in the pre-life-to-life transition on Earth.

## 6. Evolution of the Genetic Code

An appreciation of tRNA evolution appears to demand an anticodon-centric view of genetic code evolution [1,2,21]. We posit that the genetic code evolved around the tRNA anticodon according to how the anticodon was read on the coevolving ribosome. Wobbling at tRNA-34 evolved as the ribosome “learned” to read the anticodon. We posit that tRNA-34 and tRNA-36 were originally both wobble positions but that, as the code and ribosome coevolved, wobbling was suppressed at tRNA-36 by chemical modifications at tRNA-37 and by the closing of the ribosome 16S rRNA in order to tighten recognition of the tRNA–mRNA anticodon–codon interaction [55,56,57]. A comprehensive model for genetic code evolution has been published in which the placements and distributions of all amino acids in the code are rationalized according to principles of genetics and molecular biology [1,2,21]. Consistent with a polyglycine–tRNA world [37,48], glycine occupies the most favored anticodon (BCC). Consistent with a GADV–tRNA world [36,49,50,51,52,58], GADV occupies the most favored row of the code, row 4 (BNC). The model explains the evolution of related amino acids within code columns (i.e., column 1 (BAN) encodes closely related amino acids Val, Ile, Met and Leu; ValRS-IA, IleRS-IA, MetRS-IA and LeuRS-IA are closely related AARS enzymes). Features of the genetic code model include explanations for the coevolution of amino acid metabolism, amino acid chemistry, homologous AARS enzymes, tRNAomes and stop codons. 

During code evolution, previously encoded amino acids initially occupied larger segments of the code. For instance, we posit that glycine initially filled the genetic code using all available anticodons to encode glycine. To accommodate the entry of newly encoded amino acids, previously encoded amino acids retreated, retaining the most favored available anticodon positions. Favored positions in the code relate to favored anticodons according to clear preference rules. For tRNA-34 wobble, A is not utilized in Archaea [5]. Wobble tRNA-34G is favored. Also, at the base of code evolution, wobble tRNA-34C and tRNA-34U are approximately equivalent, limiting the creation of 1 codon sectors (i.e., for Met and Trp; see below). For tRNA-35 and tRNA-36, the anticodon preference rules are C > G > U >> A. So, glycine occupies the most favored anticodon (BCC). GADV occupies the most favored row 4 (BNC). Phenylalanine, tyrosine and tryptophan occupy disfavored row 1 (BNA) and are late additions to the code. Stop codons occupy disfavored row 1. The model explains why unmodified A is not utilized at a wobble position (i.e., unmodified tRNA-34A is not utilized). A rational model is proposed for serine jumping from column 2 to column 4 of the genetic code (see also [59]). Essentially, all aspects of genetic code evolution are potentially explained.

Moreover, the 1 codon sector encoding Met and Trp is explained as a somewhat special case. In Archaea, tRNA^Met^ (CAU) is utilized with minor or no wobble tRNA-34C modification to recognize Met mRNA codon AUG but not Ile codon AUA. Interestingly, tRNA^Ile^ (CAU) is also utilized but with tRNA-34C modified to agmatidine to recognize Ile codon AUA but not Met codon AUG. In the case of the tryptophan 1 codon sector, tRNA^Trp^ (CCA) shares a 2 codon box with a stop codon UGA. Stop codons are recognized by binding of protein release factors to mRNA codons [60], so no tRNA (UCA) ambiguity conflicts with the tRNA^Trp^ (CCA) reading of codon UGG.

The 6 codon sectors encoding leucine, serine and arginine have been explained. We posit that leucine, serine and arginine probably entered the genetic code at about the same time (GADVLSER world). These amino acids occupied larger segments of the code and then retreated to their current positions as new amino acids were added. Serine jumped from column 2 to column 4 of the code, as we have described. Arginine may have entered the code initially as ornithine, which was then converted to arginine through the evolution of tRNA-linked reactions. Because 6 codon sectors have previously been addressed in detail [1,2,21], the entire discussion is not reproduced here. 

Other views of genetic code evolution have been reported [61,62,63,64,65,66,67,68,69,70,71,72,73,74]. We object to codon-centric models with a complexity of 64 codons to describe the initial establishment of the code. The genetic code evolved around the tRNA anticodon and its reading on the coevolving ribosome. Wobbling at tRNA-34 limits the wobble position to purine–pyrimidine discrimination, limiting the complexity of the genetic code to 2 × 4 × 4 = 32 assignments (not 4 × 4 × 4 = 64 assignments, as in mRNA). The standard genetic code gained closure at 20 amino acids plus stops (21 assignments). Late in the process of code evolution, translational fidelity limited further expansions of the code. 

## 7. Recorded History of the Pre-Life-to-Life Transition

Sequences of tRNAs relate a history of the pre-life-to-life transition (Figure 1, Figure 2, Figure 3, Figure 4, Figure 5, Figure 6 and Figure 7). To replicate RNAs and minihelices in the pre-life world required the ligation of RNAs. The emergence of tRNAs shows that minihelices were ligated together (Figure 1 and Figure 3). The importance of RNA ligation in establishing pre-life genetic innovation and complexity cannot be overstated. Ligation of related and unrelated RNAs led to pre-life chemical and combinatorial complexity, which led to life. Ligation explains how tRNA evolved from 3 31 nt minihelices (Figure 1 and Figure 3). In order to replicate 31 nt minihelices, RNAs were ligated together, replicated and then processed to generate new minihelices. Because of side reactions of ribozymes/primitive catalysts, in the process of replicating existing RNAs, novel and more complex RNAs, such as tRNAs, were generated. The ribozymes/primitive catalysts required for these processes include the following: RNA ligases [75,76,77,78,79], RNA helicases/chaperones [80], complementary RNA template-dependent RNA replicase [81,82,83,84,85,86,87], and RNA endonucleases [88,89]. Very clearly, to support the polymer world required a ribozyme/primitive catalyst to generate and replicate RNA repeats.

Scientists have encountered some difficulties generating a ribozyme RNA template-dependent RNA replicase [87,90]. Also, to our knowledge, no one has generated a ribozyme/primitive catalyst to generate RNA repeats. These issues may be related. We suggest that the search for ribozymes/primitive catalysts to generate RNA repeats and complementary RNA covalent assemblies be broadened to determine alternate routes to support these activities. Experiments must now be performed to reproduce the polymer and minihelix world. The RNA reactants and products are known with reasonable certainty (Figure 5). Only specific ribozymes/primitive catalysts to support the transitions are lacking. Most of these ribozymes have been generated or approximated in vitro. 

We posit that complementary stems of 5 nt and 7 nt, which are found in minihelices and tRNAs, were selected because shorter stems were unstable and longer RNA duplexes, generally, were more difficult and slower to unwind using ribozyme/primitive catalyst helicases/chaperones. We posit that the tRNA U-turn loop (Figure 1, Figure 2, Figure 3, Figure 4, Figure 5 and Figure 6) was selected because it is a tight loop that resists attack by ribozyme nucleases. If a 7 nt anticodon U-turn loop has loop positions 1C and 7A (Figure 6B and Figure 7), a Hoogsteen H-bond forms that supports U-turn geometry and loop stability [46]. Once tRNA evolved, a genetic code became inevitable. Given the pre-life chemical milieu, tRNA was a molecule that could “teach” itself (“learn”) to code [1,2,21].

Carell and colleagues have indicated that modification of the 2′-O of RNA (i.e., 2′-O-methyl) may have stabilized RNA to OH^−^ hydrolysis and also may have modified ribozyme activities [38]. RNA modified at the 2′ position is expected to be a precursor to DNA. We posit that many RNA modifications existed in the pre-life world, and RNA modification enzymes coevolved with the code [91]. For instance, the evolution of the genetic code probably required modifications of tRNA-34U, tRNA-37A and tRNA-37G, at a minimum. Probably tRNA-34U methylation-based modifications were necessary to suppress “superwobbling” or 4-way wobbling in which tRNA wobble U reads mRNA wobble A, G, C and U [91,92,93]. Therefore, tRNA-34U methylation-based modifications were necessary to generate 2 codon sectors in the genetic code (i.e., column 3 of the genetic code). It appears that tRNA-37A and tRNA-37G modifications were necessary to read tRNA-36U and tRNA-36A anticodons, respectively. 

## 8. RNA Ligation, Protein Folding and Protein Pseudosymmetry

The process by which tRNA was generated in pre-life (described above) was critically dependent on RNA ligation. To replicate minihelices, RNA ligation and endonuclease processing was necessary. To generate tRNAs, RNA ligation and then processing to novel products occurred. We posit that RNA ligation was a primary mechanism for generating chemical complexity and genetic innovation in the pre-life world. Type II and type I tRNAs were clearly generated by a process in which RNA ligation played a critical role.

**Figure 8:**
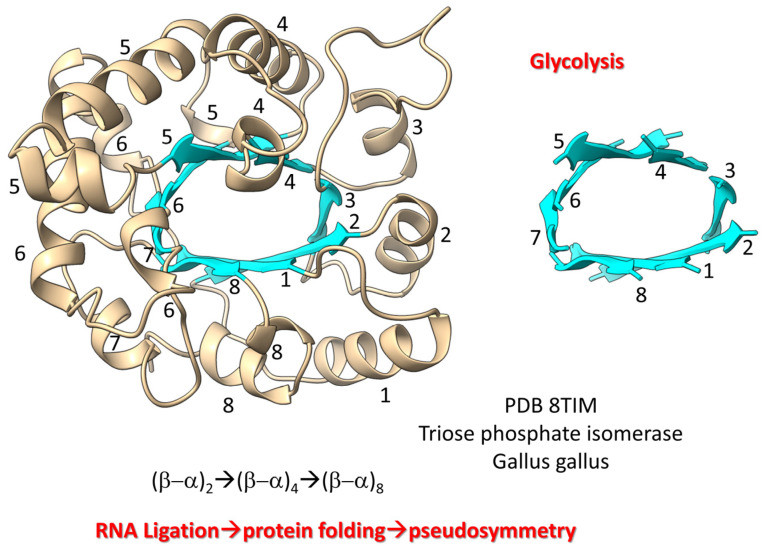
Evolution of (β−α)_8_ barrels by RNA ligation, translation and pseudosymmetrical folding. β-sheets and α-helices are numbered in the figure.

**Figure 9:**
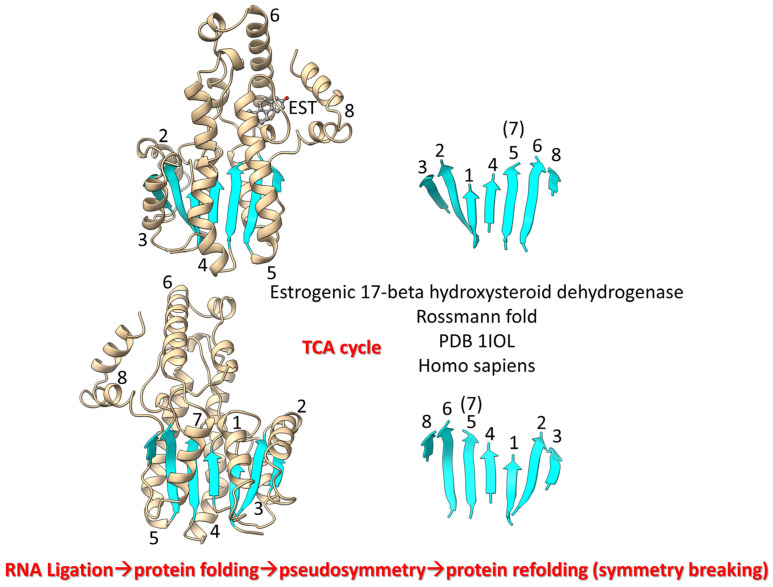
Refolding of a (β−α)_8_ barrel generated a (β−α)_8_ sheet. β7 lost its β-sheet partners in the refolding. Two views are shown. EST is estradiol. Helices are numbered in the left images. β-sheets are numbered in the right images.

**Figure 10:**
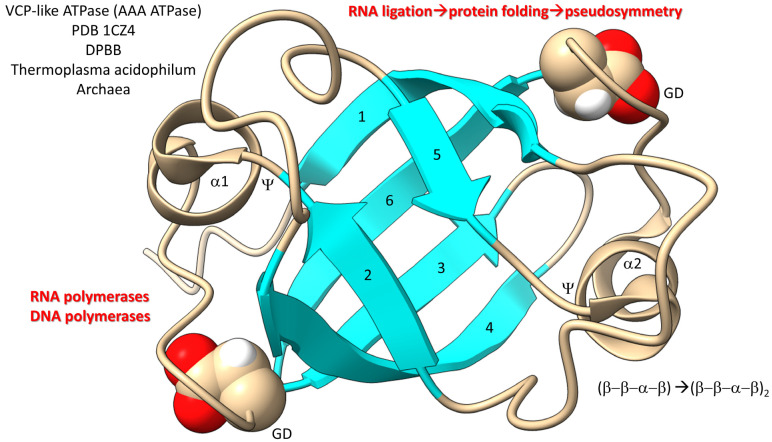
Generation of double-Ψ−β-barrels in pre-life. Numbers indicate β-sheets.

**Figure 11:**
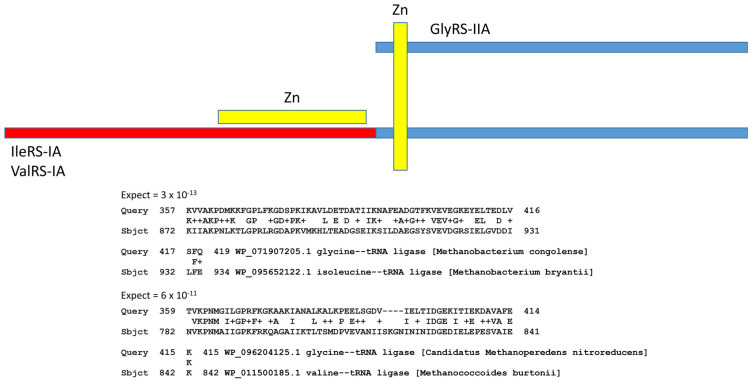
Evolution of AARS enzymes. Class II AARS are simple homologs of class I AARS. The blue segments include homologous sequences, including a Zn finger. The red segment is unique to class I AARS and directs the distinct class I AARS fold. At the bottom of the figure are two alignments demonstrating homology of GlyRS-IIA (class II), IleRS-IA (class I) and ValRS-IA (class I). + indicates amino acid similarity. Expect values indicate homology of sequences.

The first proteins (i.e., ribosomal proteins, AARS enzymes, (β−α)_8_ barrels (i.e., TIM barrels), (β−α)_8_ sheets (Rossmann folds), RNA modification enzymes, RNA and DNA polymerases) coevolved with the genetic code [94]. We posit that from about the 8 amino acid stage of genetic code evolution, sequence-dependent proteins began to coevolve with the emerging code. We posit that RNA ligation was a central feature in the evolution of the first proteins. As examples, we show Figure 8, Figure 9, Figure 10 and Figure 11. Figure 8 shows a (β−α)_8_ barrel protein (i.e., a TIM barrel protein; TIM for triose phosphate isomerase). Because β-sheets require a partner β-sheet, we posit that (β−α)_8_ barrels were generated by ligation of two (β−α)_2_ RNAs encoding parallel β-sheets to form a (β−α)_4_ RNA encoding all parallel β-sheets. Ligation of two (β−α)_4_ RNAs created a (β−α)_8_ RNA encoding all parallel β-sheets that was translated and folded into a (β−α)_8_ barrel [95]. In pre-life, folding proteins into pseudosymmetrical barrels may have resulted from RNA ligations combining multiple identical RNAs. Translation of RNA repeats generated protein repeats that could fold into barrels. Glycolytic enzymes include (β−α)_8_ barrels, so we have described the evolution of glycolysis in pre-life.

We posit that during pre-life (β−α)_8_ barrels were rearranged into (β−α)_8_ sheets (Rossmann folds) by protein refolding (Figure 9) [96]. TCA cycle enzymes include Rossmann folds. Thus, significant metabolic capacity coevolved with the genetic code in pre-life via RNA ligations, much as described for the evolution of type II and type I tRNAs. 

In Figure 10, a domain of a AAA-ATPase is shown [97,98,99]. This pseudosymmetrical barrel is a double-Ψ−β-barrel (β−β−α−β)_2_. We posit that the double-Ψ−β-barrel was formed from ligation of two (β−β−α−β) RNAs followed by translation and pseudosymmetrical folding. PolD (an archaeal DNA polymerase) and multi-subunit RNA polymerases are 2 double-Ψ−β-barrel polymerases. The GD motif in the loop separating β5 and β6 was the precursor to the NADFDGD motif that is highly conserved in multi-subunit RNA polymerases [100,101]. We posit that these core life functions were generated in pre-life via RNA ligation, translation and pseudosymmetrical folding. Thus, based on the apparent RNA ligation mechanism for tRNA evolution, glycolysis, the TCA cycle, AAA-ATPases, DNA polymerases and RNA polymerases may have been generated during pre-life [102].

AARS enzymes coevolved with tRNAomes and the genetic code. The evolution of AARS enzymes, however, has been improperly understood [1,2,22]. We have attempted to clarify how AARS enzymes evolved. Despite their different folds, class II and class I AARS enzymes are simple homologs (Figure 11). Notably, GlyRS-IIA is a homolog of IleRS-IA and ValRS-IA. In ancient Archaea, these enzymes often share a Zn finger and significant homology. At the bottom of the figure, we show local alignments of the same segment of GlyRS-IIA with homologous segments of IleRS-IA and ValRS-IA. Initially, class II and class I AARS were not thought to be homologous because these enzymes have distinct folds. We posit that, in pre-life, a primitive ValRS-IA was derived from a primitive GlyRS-IIA by ligation of a distinct RNA encoding the ValRS-IA N-terminal segment, followed by translation and folding. The addition of the N-terminal ValRS-IA extension determined the distinct class I AARS fold, as we have described.

## 9. Proof of the 3 31 nt Minihelix tRNA Evolution Theorem

The axioms (i.e., assumptions; obvious statements of proposition) on which the proof is based are as follows: (1) an obvious GCG repeat (5′ acceptor stem) is a GCG repeat (Figure 7); (2) a GCG repeat complement, a CGC repeat (3′ acceptor stem) is a CGC repeat; (3) a UAGCC repeat is a UAGCC repeat; and (4) a homologous anticodon (CCGGG_CU/???AA_CCCGG) 17 nt stem-loop-stem and a T (CCGGG_UU/CAAAU_CCCGG) 17 nt stem-loop-stem are homologous stem-loop-stems [1,2,3,21]. The theorem has made the following predictions: (1) the statistics reported in Table 1 were predicted [3,24,42] and (2) the type II V loop was initially based on the ligation of a 7 nt 3′-acceptor stem and a 7 nt 5′-acceptor stem (Figure 1 and Table 1) [42]. The very few sequence deviations from the theorem are readily justified by principles of tRNA folding. For instance, G19 was selected to be G instead of A (in the third D loop UAGCC repeat) to support D loop–T loop interactions at the elbow of tRNA (Figure 7) [3]. Minor sequence changes in the D stem and V loop occurred to support the tRNA fold versus a minihelix fold (i.e., the Levitt base pair in type II tRNAs). The theorem predicts and justifies all sequences found in tRNAs of Archaea and Bacteria. This is a paper in which definite and publically available conserved tRNA sequence data were used to reach definite conclusions about the origin of life on Earth.

## 10. Discussion and Conclusions

In the context of pre-life, tRNA was a molecule that could teach itself to code. Because tRNA^Pri^ sequences were highly patterned and their order conserved, the evolution of type II and type I tRNAs is a solved problem. The tRNAomes of LUCA and organisms diverged from type II and type I tRNAs by duplications, mutations and repurposing. For obvious reasons (see above), no chaotic convergent or accretion model can be reasonable to describe the earliest tRNA evolution. To build the genetic code, the evolution of mRNA codons conformed to the evolution of tRNAome anticodons. 

The genetic code coevolved with amino acid metabolism, polypeptides, tRNAomes, mRNA, AARS enzymes, first proteins and enzymes, tRNA modification enzymes (some of which depend on relics of (β−α)_8_ barrels) [103] and protocells. The patterns of relatedness within tRNAomes and AARS in ancient Archaea relate to a history of the evolution of the genetic code. We posit that RNA ligation was a major driving force for the evolution of the complexity of the first RNAs and the folding complexity and pseudosymmetry of the first proteins. 

Sequences of tRNAs are among the most highly conserved from pre-life. Sequences of rRNA have been reported to have been derived, in part, from tRNA sequences [29,30,31,32]. The first proteins appear to include sequences and structures that partly reflect the pre-life-to-life transition (Figure 8, Figure 9, Figure 10 and Figure 11). So far as we are aware, tRNA sequences represent the clearest insights into the pre-life-to-life transition on Earth. 

To teach the biological sciences, integrate and emphasize genetic coding, evolution, sequence analysis, translation, structure and the central functions of RNA in biology. The history of tRNA chemical evolution, as written into and conserved in living genetic code, relates these fundamental lessons and describes the pre-life-to-life transition from about 4 billion years ago. Note that we are advocating for a tRNA-centered evolution of life on Earth based on analysis of tRNA sequences conserved over about 4 billion years since life began. The RNA world was initially hypothesized for similar reasons because tRNA, mRNA and rRNA form the functional core of molecular biology. The concept was to identify core conserved functions and to evolve around them toward more complex systems. We advocate for centering attention on tRNA. It appears to us that mRNA and rRNA coevolved to accommodate the central functions of tRNAs in the evolution of coding. The evolution of tRNA combines aspects of evolution, the birth of biology on Earth, the origin of life, chemical evolution, sequence pattern recognition, coding and decoding, tRNA modifications, learning and genetic memory and the evolution of the patterning of the genetic code. A tRNA-first approach to the evolution of the genetic code makes sense. Ribosome-first, mRNA-first and genetic code-first approaches are illogical. 

Because tRNA evolution was first solved by inspection and as a puzzle, tRNA evolution should be of interest to gamers and puzzlers. Because tRNA evolution is a problem in biological coding, learning and problem-solving, tRNA evolution is a subject for programmers and psychologists. Linguists and mathematicians have shown an interest in the evolution of the genetic code [68,69,70,71,72,73,74]. Sentient humans on Earth must know this content.

TRNA forms the functional core of living systems on Earth. The ribosome apparently evolved largely after tRNA. For instance, the ribosome includes a tRNA-shaped channel through the 23S rRNA within the large ribosomal subunit [104]. Also, rRNA is hypothesized to have evolved partly from tRNA fragments [29,30,31,32]. The anticodon 7 nt U-turn loop was selected in pre-life to support a 3 nt genetic code [1,2,3,21,24]. We posit that once tRNA chemically evolved, the evolution of the genetic code became inevitable. Acceptance of these ideas allows pre-life on Earth to be described. The rejection of these ideas is not useful in describing the chemical evolution of life on Earth.

On Earth, tRNA is the genetic adapter. Without a genetic adapter, there can be no genetic code and no life. To re-engineer tRNA to form a different genetic adapter would be problematic. To design a genetic adapter using another chemistry than RNA seems unlikely. To redesign the anticodon loop of tRNA would present a significant challenge (Figure 6B). tRNA evolution presents a remarkable history of the pre-life-to-life transition on Earth. If life is discovered on Enceladus (or elsewhere in the cosmos), we posit it will utilize a similar adapter molecule to tRNA. tRNA and the tRNA anticodon loop are core intellectual properties in the evolution of life.

## Figures and Tables

**Table 1:** Internal homologies within archaeal tRNAs. C indicates a sequence complement. In type II V loops, n indicates the total length of the V loop. In type II tRNAs, V_1_ was selected to be U to form a G26~UV_1_ wobble pair. V_n_ was selected to be C to form the G15-CV_n_ reverse Watson–Crick Levitt base pair. Similar statistics were obtained for Bacterial tRNAs [24,42].

Sequence #1	Sequence #2	Length (nt)	*p*-Value
5′-As	3′-As-C	7	0.001
3′-As	5′-As-C	7	0.001
5′-As*	5′-As (3 to 7)	5	0.001
3′-As* (V loop)	3′-As (66 to 70)	5	0.001
Ac SLS	T SLS	17	0.001
D loop	Ac loop	17	0.979
D loop	T loop	17	~1
3′-As and 5′-As (66 to 71 and 2 to 7)	V-loop Leu V_2_ to V_7_ and V_n-7_ to V_n-1_	12	0.001
3′-As and 5′-As (66 to 71 and 2 to 7)	V-loop Ser V_2_ to V_7_ and V_n-7_ to V_n-1_	12	0.001

## Data Availability

Not applicable.
